# Supplementary material for: A robust spike sorting method based on the joint optimization of linear discrimination analysis and density peaks
Source: Sci Rep. 2022 Sep 15;12:15504. doi: 10.1038/s41598-022-19771-8 (PMC9477889; doi:10.1038/s41598-022-19771-8)
Supplement: Supplementary file 1 — Supplementary Figures. [file 41598_2022_19771_MOESM1_ESM.docx]

**Supplementary**

**A Robust Spike Sorting Method based on the Joint Optimization of Linear Discrimination Analysis and Density Peaks**

**Yiwei Zhang**^1^**, Jiawei Han**^1,2^**, Tengjun Liu**^1^**, Zelan Yang**^1^**, Weidong Chen**^1,3,*^**, Shaomin Zhang**^1^


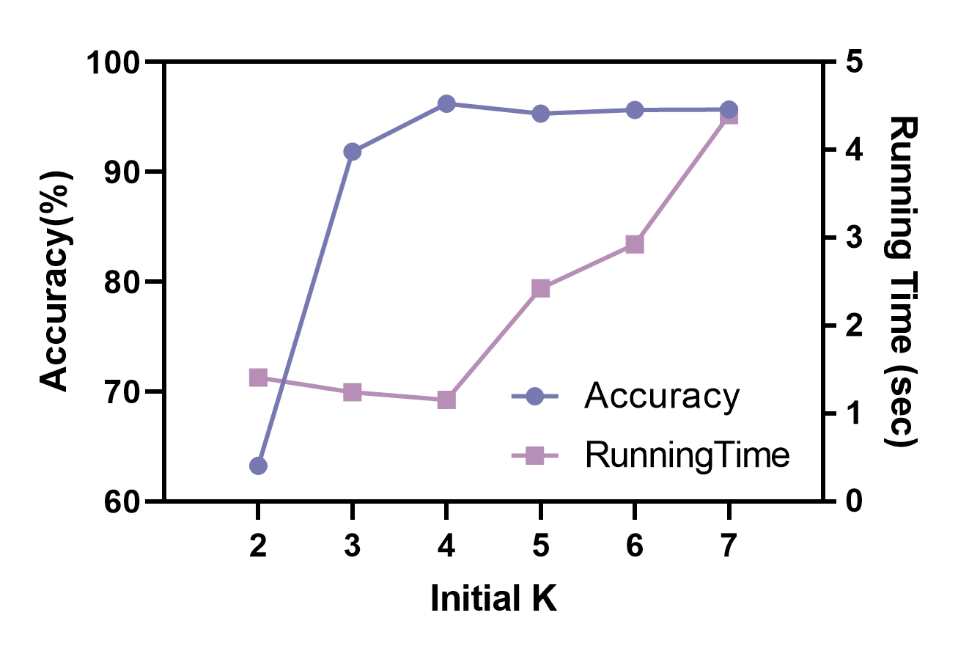


**Figure S1.** The average sorting accuracy and running time of our LDA-DP with respect to the value of initial $K$. We evaluated the performance on all 20 testing sets in Dataset A. It is notably that when $K=4$, the average accuracy reaches a plateau, with the average running time remains at a low level.


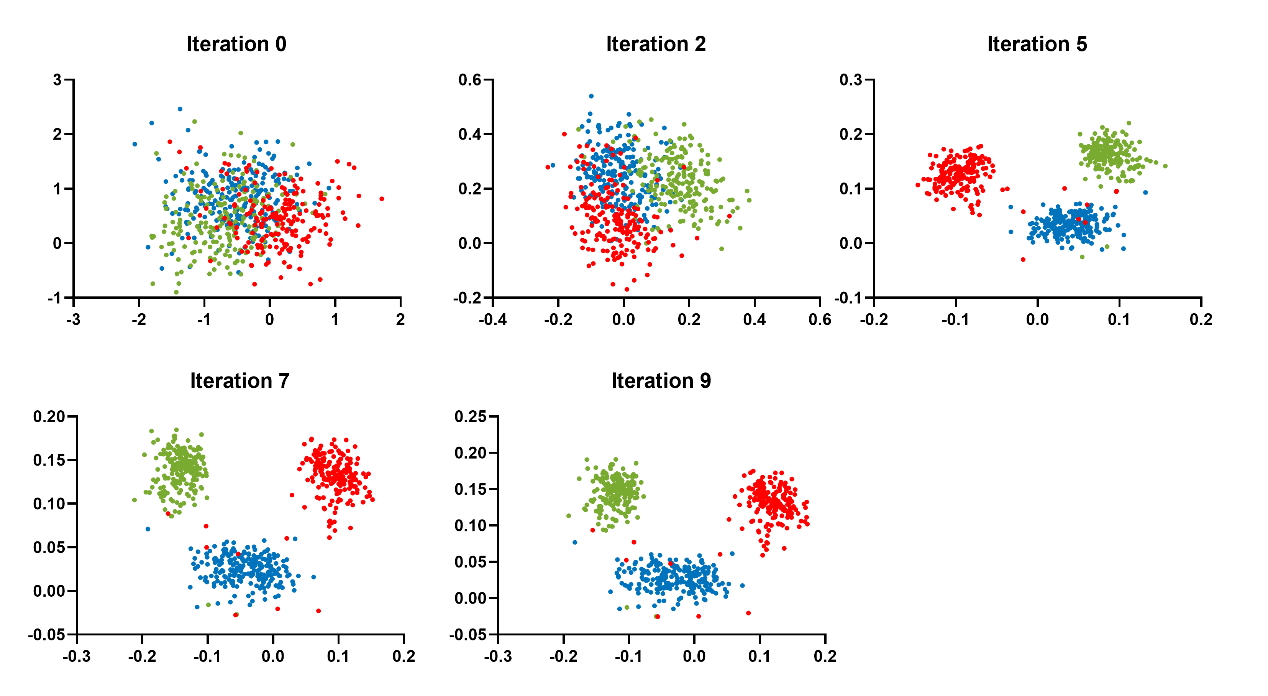


**Figure S2.** Two-dimensional features in each iteration of the LDA-DP on testing set C3_020. The data points were colored according to the ground truth. Red, green and blue represent the features from three clusters, respectively.

**
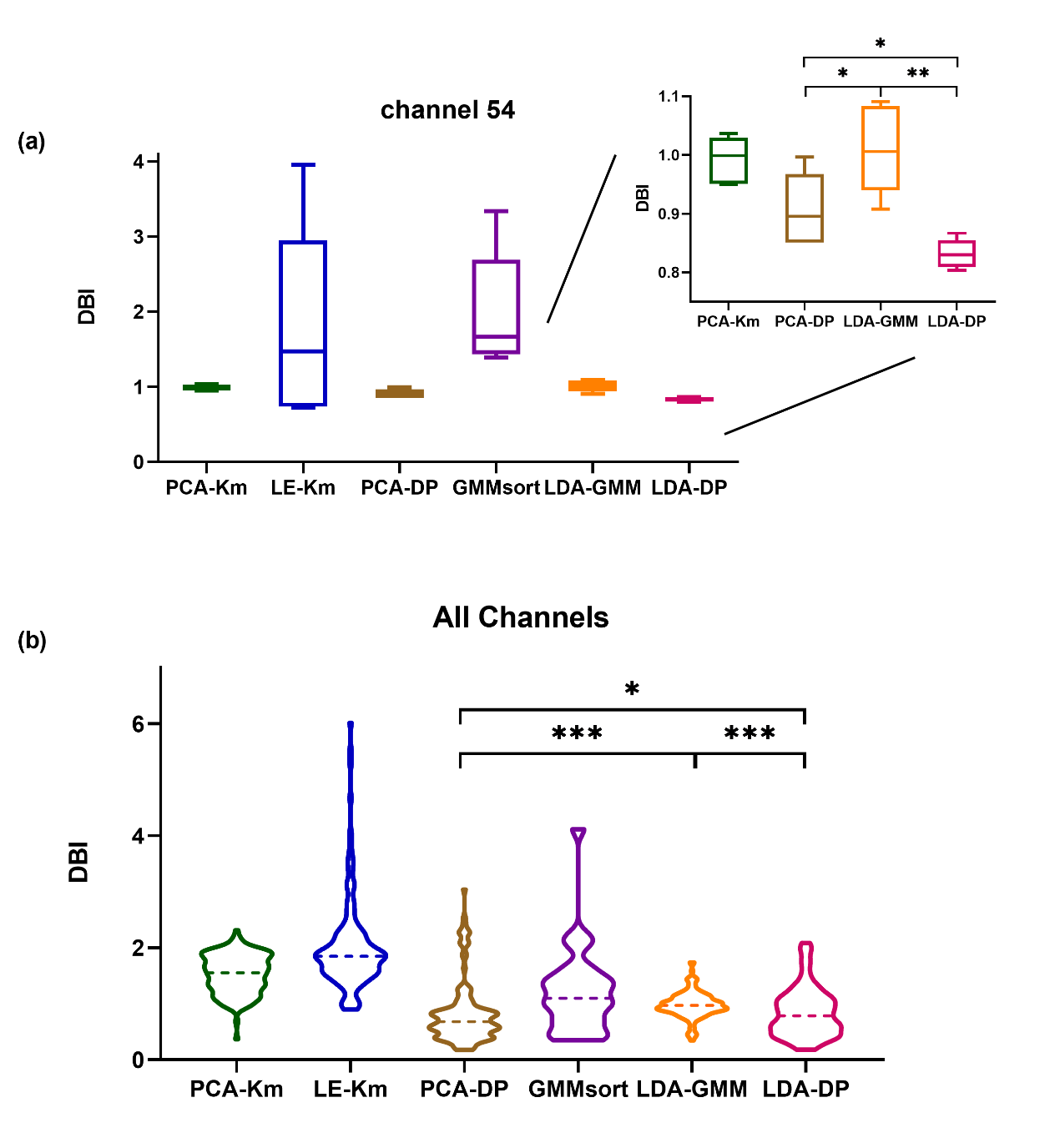
**

**Figure S3.** The sorting results on Dataset C when comparing 6 spike sorting algorithms. (a) Comparison of cluster quality using spikes from one particular channel 54 in dataset C. We conducted a 5-fold cross-validation and draw boxplots of the DBI index for 6 algorithms. Inset: boxplot of the DBI index for PCA-Km, PCA-DP, LDA-GMM and LDA-DP. * p<0.05, ** p<0.01, Kruskal-Wallis test. (b) Comparison of cluster quality using spikes from all 30 channels in dataset C. We conducted a 5-fold cross-validation for each channel and then drew the violin plot. The dashed lines represent the median of the DBI. * p<0.05, *** p<0.001, Kruskal-Wallis test.


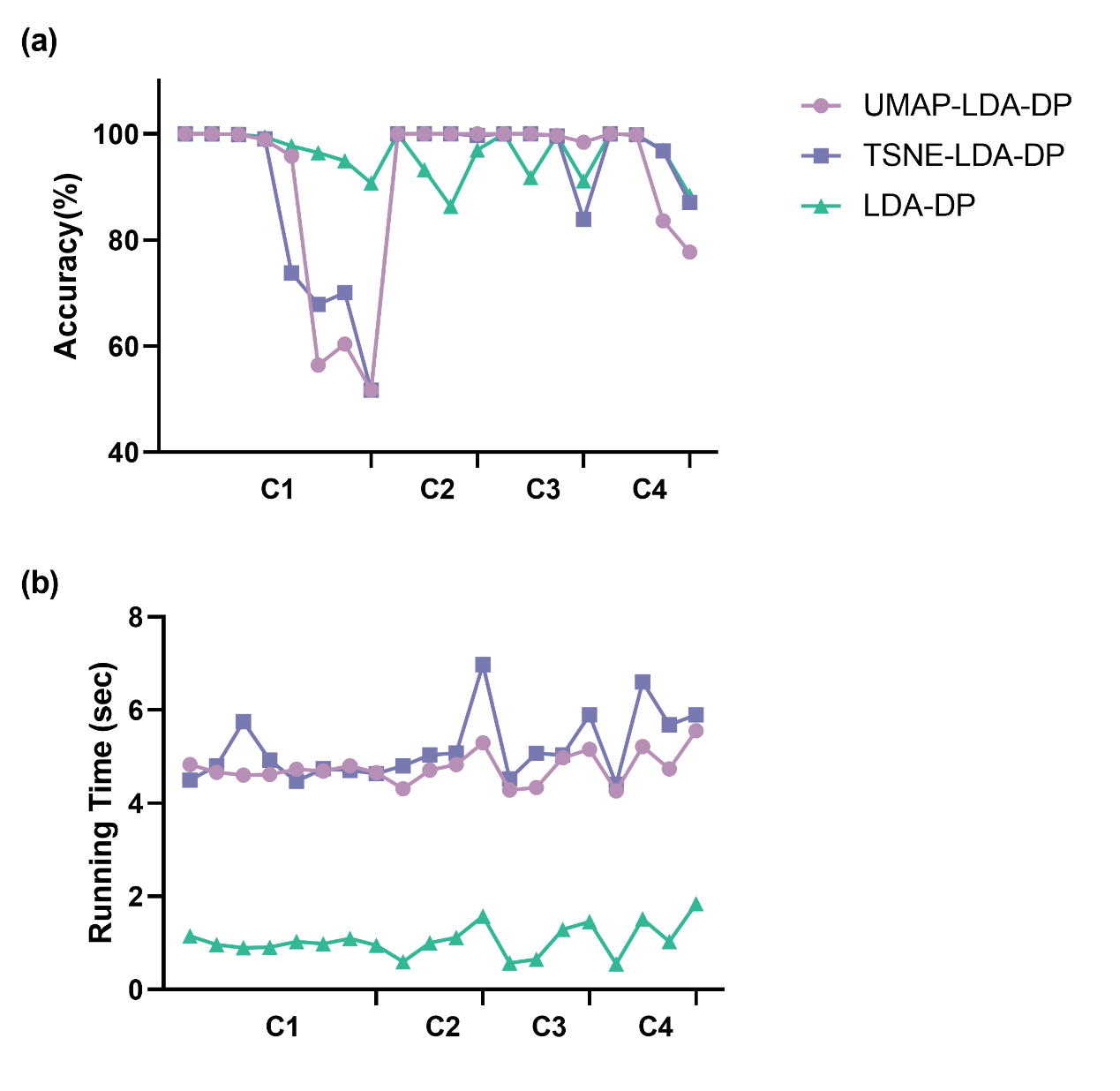


**Figure S4.** Comparison of 3 algorithms on Dataset A. UMAP-LDA-DP, TSNE-LDA-DP and LDA-DP represent 3 different algorithms using UMAP, TSNE and PCA as the initial method, respectively. (a) Accuracy. (b) Running time.


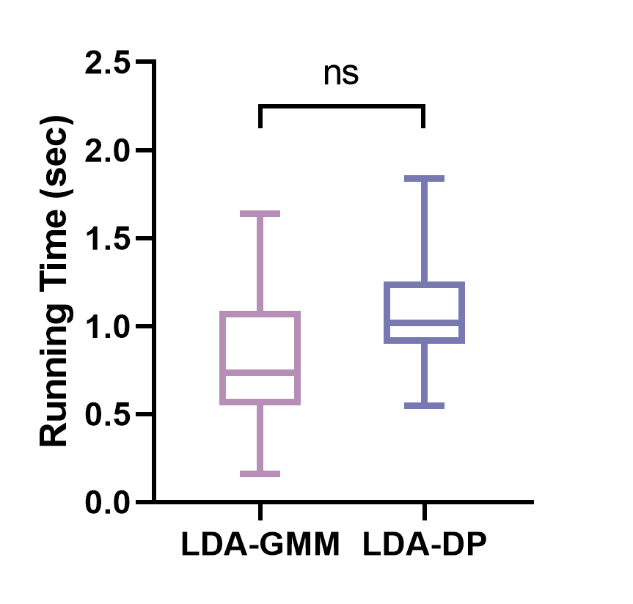


**Figure S5.** Running time comparison of LDA-GMM and LDA-DP on Dataset A. ns represents not significant, paired t-test, p=0.11 > 0.05.


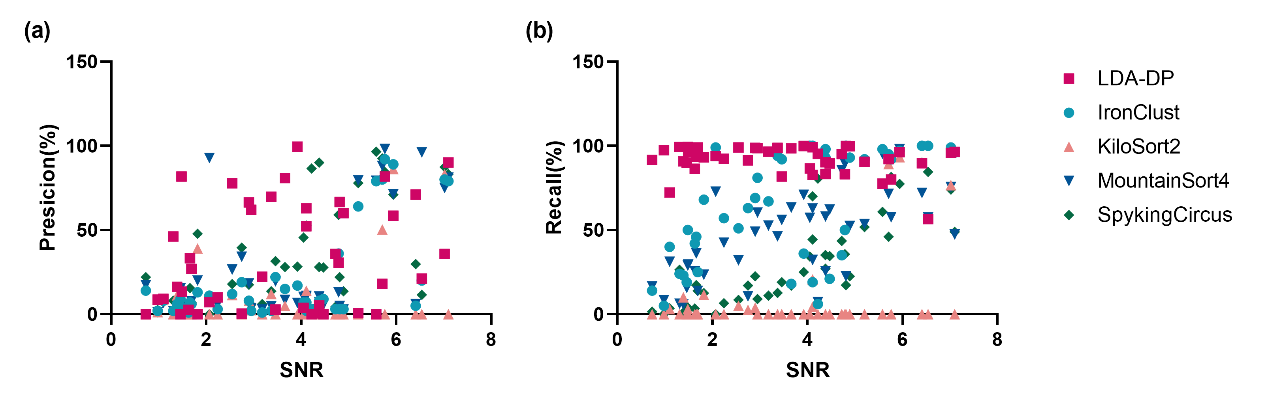


**Figure S6.** Performance comparison between the LDA-DP and the other 4 algorithms in SpikeForest on in-vivo Dataset B, with regard to SNR. (a) Precision. (b) Recall.
